# WhatsApp-Led mHealth Intervention for Hypertension Management Among Adults Aged 40 to 59 Years in Kerala, India: Protocol for a Mixed Methods Study

**DOI:** 10.2196/81307

**Published:** 2026-04-20

**Authors:** Karthika Maniyara, Prakash Babu Kodali

**Affiliations:** 1 Department of Public Health and Community Medicine, Central University of Kerala, Tejaswini Hills, Periya, Kasaragod, 671320, India, 91 8330963085; 2 Center for Tobacco Control Research and Education, University of California, San Francisco, San Francisco, CA, United States

**Keywords:** adherence, hypertension, lifestyle, medication, mobile health, mHealth, WhatsApp

## Abstract

**Background:**

Medication adherence and lifestyle modification remain key in the management of hypertension. Leveraging mobile technologies to improve patient adherence has been found to be effective in chronic disease management. Given the high mobile penetration and the opportunities it provides for encouraging lifestyle modification, an app such as WhatsApp, which is a free-to-use and real-time messaging platform, could facilitate patient-clinician interactions. We present a protocol for a WhatsApp-led mobile health (mHealth) intervention to improve adherence to medication and healthy lifestyles among adults aged 40 to 59 years in Kerala, India.

**Objective:**

The study aims to assess the impact of the WhatsApp-led mHealth strategy on blood pressure measures and adherence to medication and lifestyle modification. The study also focuses on exploring participants’ views on feasibility, facilitators, and challenges, as well as their intention to use this WhatsApp-led mHealth strategy.

**Methods:**

The study will follow a multiphase mixed methods (sequential explanatory) design with an initial quantitative phase (quasi-experimental study design) followed by a qualitative phase (phenomenological approach). Multistage cluster sampling will be adopted. Using a survey checklist and blood pressure measurements, community-dwelling adults aged 40 to 59 years will be screened for hypertension. Approximately 120 participants with hypertension will be assigned each to the intervention and control arms. All the participants will be advised to follow routine hypertension care from health care providers. Additionally, participants in the intervention arm will receive regular health messages and reminders over WhatsApp. A baseline survey will be conducted at enrollment, and a follow-up survey will be conducted after 3 months at the end of the intervention. The qualitative phase will involve follow-up interviews. Participants will be selected through quota sampling from those with the lowest and highest adherence to medication and lifestyle modification after receiving the intervention. Statistical analysis for survey data and thematic analysis for qualitative data will be conducted.

**Results:**

The study received ethics approval (IHEC/CUK/2024/04) from the Institutional Human Ethics Committee of the Central University of Kerala. All 240 participants were recruited by July 2025. The study completed data collection in August 2025 and is expected to finish data analysis by April 2026. The study results are expected to be submitted for publication in July 2026.

**Conclusions:**

The study will be a potential strategy to address nonadherence to treatment and lifestyle regimens, which is a critical bottleneck to controlling hypertension and preventing complications. The study could be proof for a WhatsApp-led mHealth strategy that can be integrated into hypertension management at the community level, especially for improving hypertension awareness and reminders for behavior change.

## Introduction

Increased blood pressure levels are considered a prominent risk factor for cardiovascular diseases, which account for one-third of the deaths in India [1]. Uncontrolled hypertension is associated with nonadherence to medication and healthy lifestyle practices [2,3]. For the management of chronic conditions such as hypertension, India’s National Health Policy 2017 encourages the deployment of digital technologies to supplement routine health care. Digital technologies for hypertension management have been shown to promote treatment adherence and encourage lifestyle modification (ie, increased physical activity and better diet) [4-9].

Adults in the age group of 40 to 59 years are a crucial demographic for hypertension management. They have a greater risk of developing hypertension than their younger counterparts and better odds of controlling blood pressure compared to individuals older than 60 years. Major bottlenecks for hypertension management and control in this group lie in lifestyle modification and adherence to medication. Given the high mobile penetration, better digital literacy, and the opportunities for encouraging lifestyle modification that they provide, mobile-based interventions could be particularly effective among this population group for facilitating chronic disease management. WhatsApp, as a free-to-use and cost-effective app with better ease of use and user acceptance, has been increasingly used in organizational communication, monitoring, and delivery of services. As a real-time messaging platform, it has been shown to facilitate patient-clinician interactions [10,11]. Instant messaging through WhatsApp can facilitate mobile health (mHealth) services by acting as a medium for communicating reminders, health advice, and hypertension management guidelines [5,6,9,12,13].

A significant challenge of disease-specific mHealth apps is limited acceptance and ease of use among end users (patients). Given that hypertension management includes adherence to medicine and lifestyle changes, behavior change communication through WhatsApp-mediated reminders could function as cues to action and subsequently promote hypertension management. The effectiveness and feasibility of such an intervention are yet to be explored.

**Figure 1.**
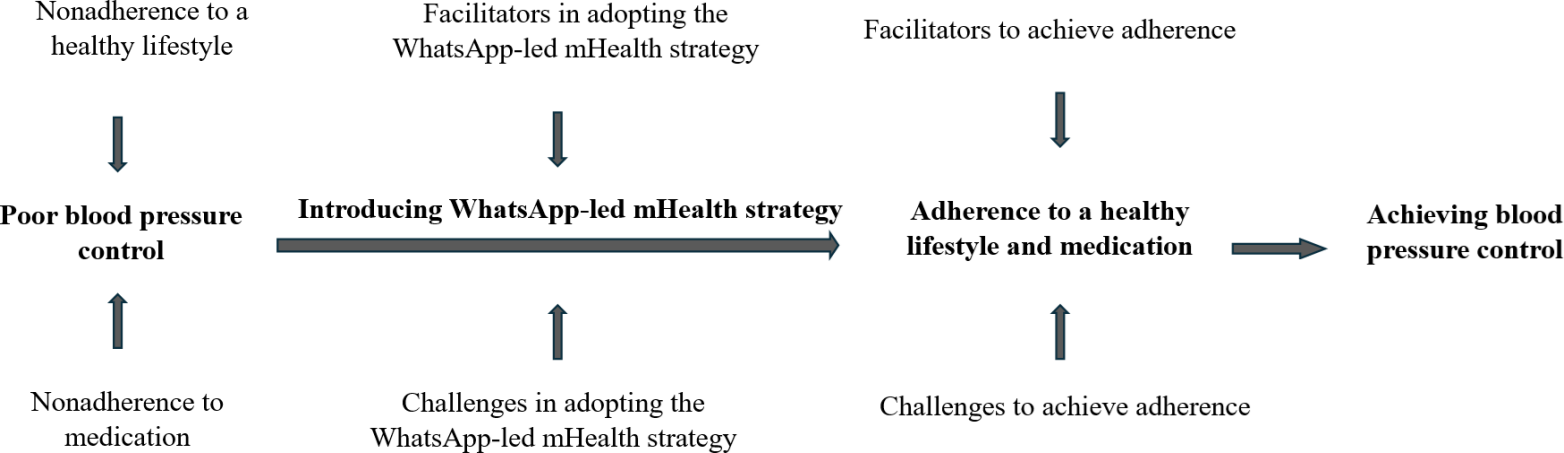
Conceptual framework for the study. Clinically, hypertension control is achieved when systolic blood pressure is below 140 mm Hg and diastolic blood pressure is below 90 mm Hg as a result of lifestyle modification or medication [14]. mHealth: mobile health.

In this study, we aim to assess the impact of a WhatsApp-led mHealth strategy on blood pressure measures and adherence to medication and lifestyle modification among adults with hypertension aged between 40 and 59 years. The study also explores the participants’ intention to use the mHealth strategy and their views on the feasibility, facilitators, and challenges of using a WhatsApp-led mHealth strategy for adhering to medication and a healthy lifestyle for hypertension management. The conceptual framework adopted for the study (Figure 1 [14]) explains the facilitators and challenges of adopting the mHealth strategy for medication and lifestyle adherence using the Andersen-Newman behavioral model of health service use, unified theory of acceptance and use of technology, and health belief model [15-17].

## Methods

### Study Design and Protocol

The study will adopt a multiphase mixed methods approach with a sequential explanatory design: an initial quantitative phase followed by a qualitative phase. The study protocol follows the Mixed Methods Reporting in Rehabilitation and Health Sciences guidelines, mHealth Evidence Reporting and Assessment checklist, and Transparent Reporting of Evaluations with Nonrandomized Designs statement [18-20].

### Study Setting and Population

Given the high prevalence and low control rates of hypertension in the Malabar region of Kerala, India, especially in Kannur [21,22], the study will be conducted among adults aged 40 to 59 years in the Kannur district, Kerala.

### Quantitative Phase of the Study

#### Study Design

The quantitative phase will use a quasi-experimental study design with an intervention group and a control group. This phase is designed to assess the effectiveness of the WhatsApp-led mHealth intervention on blood pressure estimates and medication and lifestyle adherence among individuals with hypertension.

#### Sample Size

A total study sample size of 240, with 120 each in the intervention and control arms, was estimated based on the estimates of diastolic blood pressure at baseline (mean 80.9, SD 10.9 mm Hg) and the postintervention time point (mean 75.4, SD 11.5 mm Hg) from a previous randomized controlled trial in Kerala [23]. *OpenEpi* was used for sample size calculation. The sample size was estimated at 80% power, a 95% CI, a 20% nonresponse rate, and a design effect of 1.5.

#### Sampling

**Figure 2.**
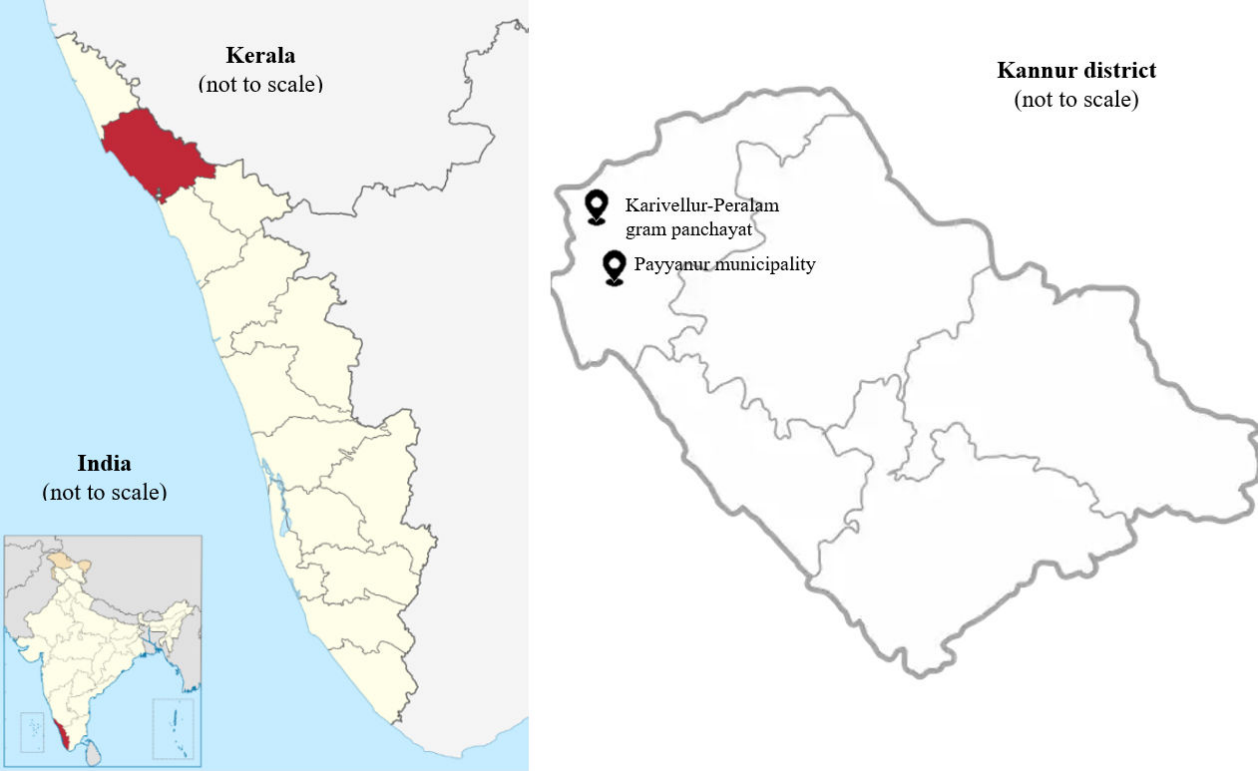
Study location in the Kannur district of Kerala, India. Source: Wikimedia Commons (2023).

**Figure 3.**
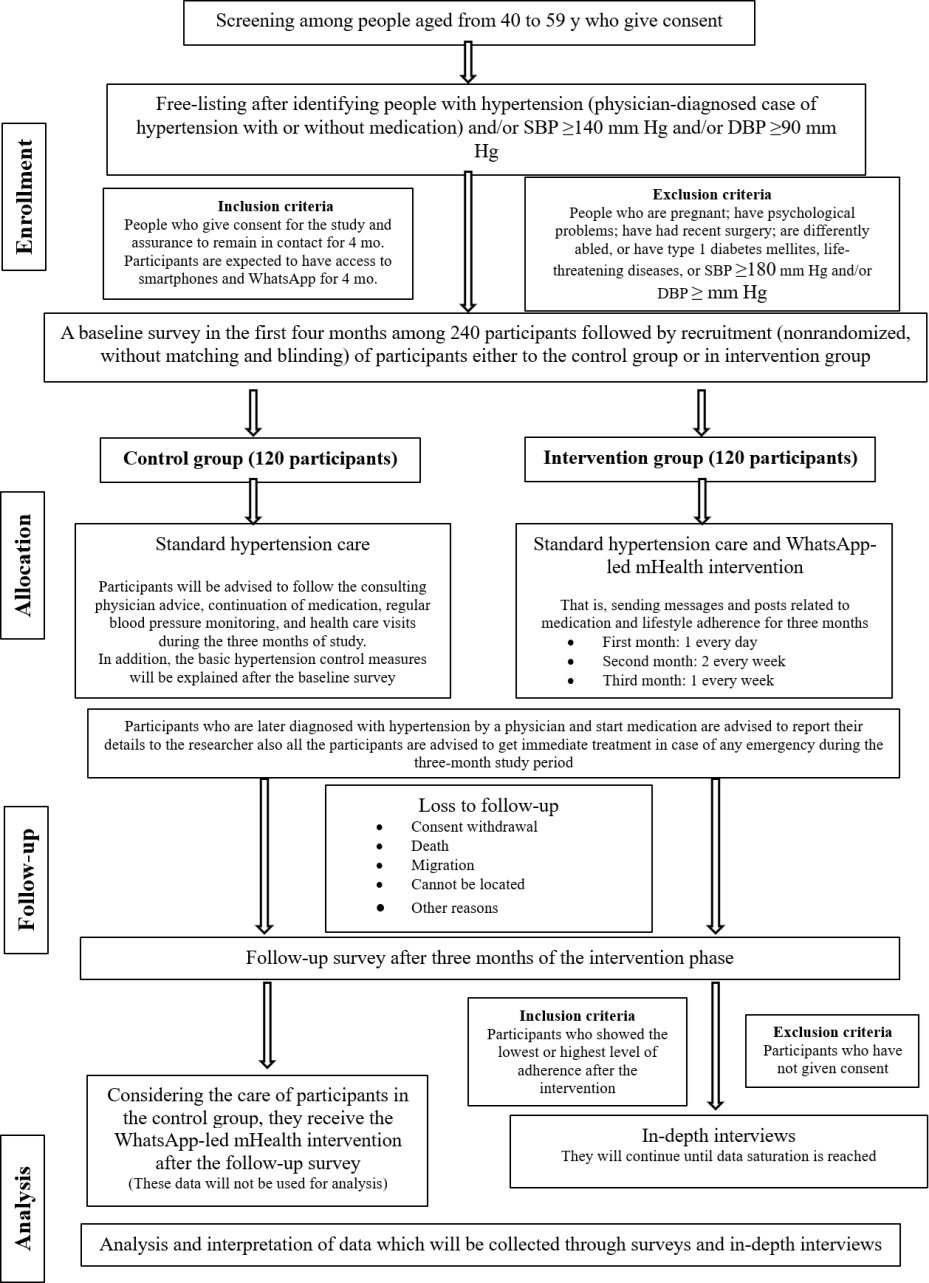
Transparent Reporting of Evaluations With Nonrandomized Designs (TREND) statement flow diagram of the study. DBP: diastolic blood pressure; mHealth: mobile health; SBP: systolic blood pressure.

The study followed a multistage cluster sampling method. In the first stage, all taluks in the Kannur district were listed, and then 1 taluk (ie, Payyanur taluk) was selected. One rural (ie, Karivellur-Peralam gram panchayat) and one urban (ie, Payyanur municipality) administrative unit (cluster) from this taluk were then selected for the study (Figure 2). In stage 2, a door-to-door household survey will be conducted in these 2 rural and urban clusters. Household members aged 40 to 59 years will be screened for eligibility after consent is obtained. In the third stage, eligible participants (those with hypertension and access to WhatsApp) will be surveyed. After the baseline survey, the study’s recruitment will follow a nonrandomized approach (Figure 3) without matching or blinding as these are not applicable in this study setting. Upon recruitment and consent, the first participant will be allocated to the intervention group, and the next participant will be allocated to the control group. The participants will be recruited into the intervention and control groups sequentially in a 1:1 ratio. This process will be conducted iteratively until the sample size of 240 is reached (ie, 120 in the intervention group and 120 in the control group). At the cluster level, this amounts to 120 participants per cluster (urban and rural), with 60 of them in the intervention group and 60 in the control group.

#### Standard Hypertension Care

All participants will be advised to follow the consulting physician’s advice, continue medication, maintain regular blood pressure monitoring, and attend health care visits. Participants will receive health education, including the importance of healthy eating with controlled salt intake and adequate fruit and vegetable intake; regular physical activity; proper medication; and avoiding unhealthy behaviors such as alcohol consumption, tobacco use, and processed food intake. Participants will be reminded about the follow-up survey, which will be conducted 3 months after the baseline survey.

#### Intervention*:* WhatsApp-Led mHealth Strategy

**Figure 4.**
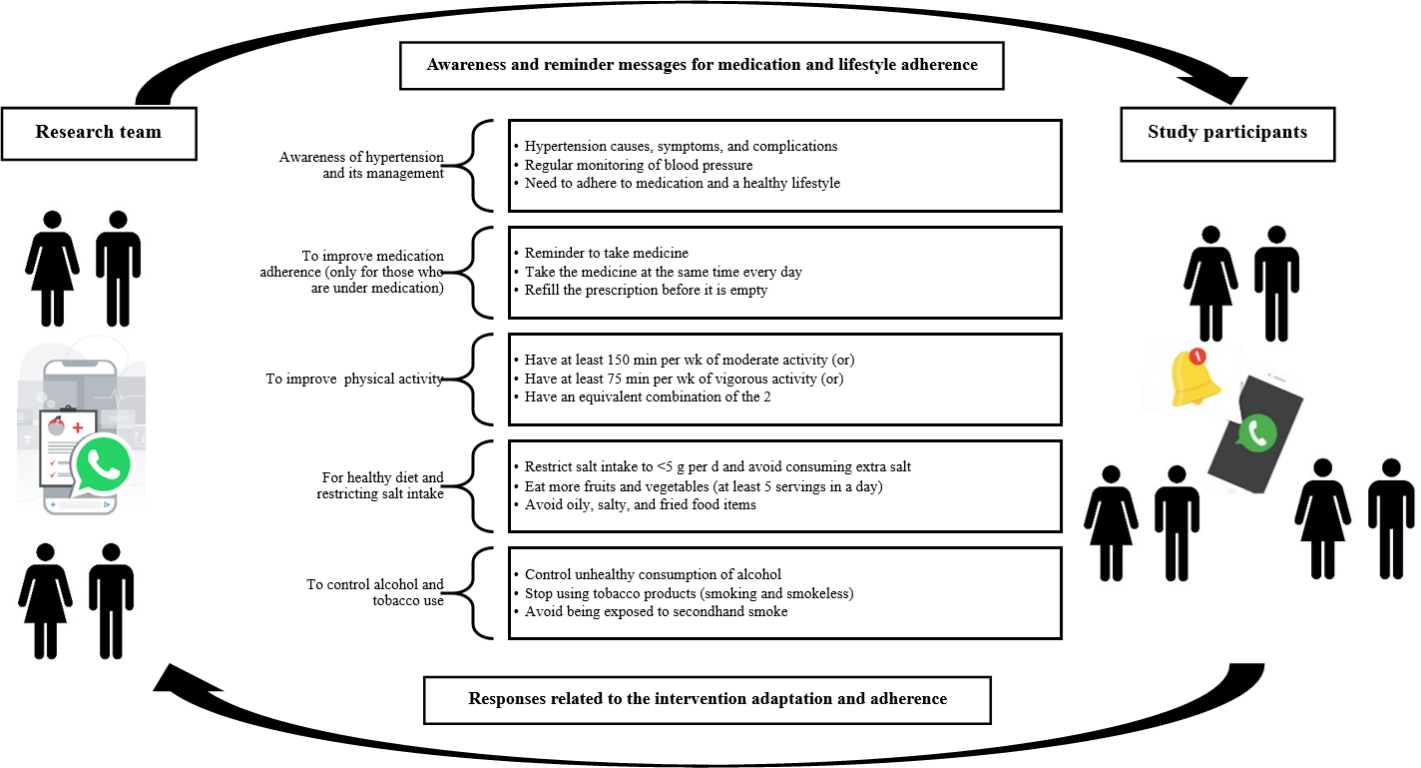
WhatsApp-led mobile health intervention for medication and lifestyle adherence in participants with hypertension.

A WhatsApp-led mHealth strategy was developed (Figure 4) with participant-specific messages and posters, which were prepared based on standard hypertension management protocols [24-28]. WhatsApp, an instant messaging platform owned by technology conglomerate Meta Platforms, was chosen due to its universal adoption and ease of use and the possibility of sending health messages through text and multimedia (image, voice, and video) formats. The intervention duration was determined to be 3 months based on the review period for hypertension as per the hypertension management protocol of the Department of Health Services, Government of Kerala [12,24].

The diagnosis and treatment details of the study participants will be obtained during the baseline survey. Furthermore, the messages will be tailored considering their hypertension treatment status (ie, those who are under medication and those who are not under medication). Among those who are under medication, awareness of healthy lifestyles will be promoted, and they will receive reminders for proper medication adherence, regular consultation with their treating physician, and blood pressure monitoring. Newly diagnosed individuals with hypertension and those who are not in treatment will be sent health messages highlighting the need to adhere to positive health behaviors, consult a physician, and monitor their blood pressure periodically.

The messages for a healthy lifestyle include awareness and reminder messages for keeping healthy eating habits, focusing on the Dietary Approaches to Stop Hypertension diet. The messages also include the need for adequate physical activity and a list of physical activities classified by effort level (mild, moderate, and vigorous). Messages on the importance of reducing mental stress, quitting smoking, avoiding exposure to secondhand smoke, and controlling alcohol intake will also be shared with the participants.

**Figure 5.**
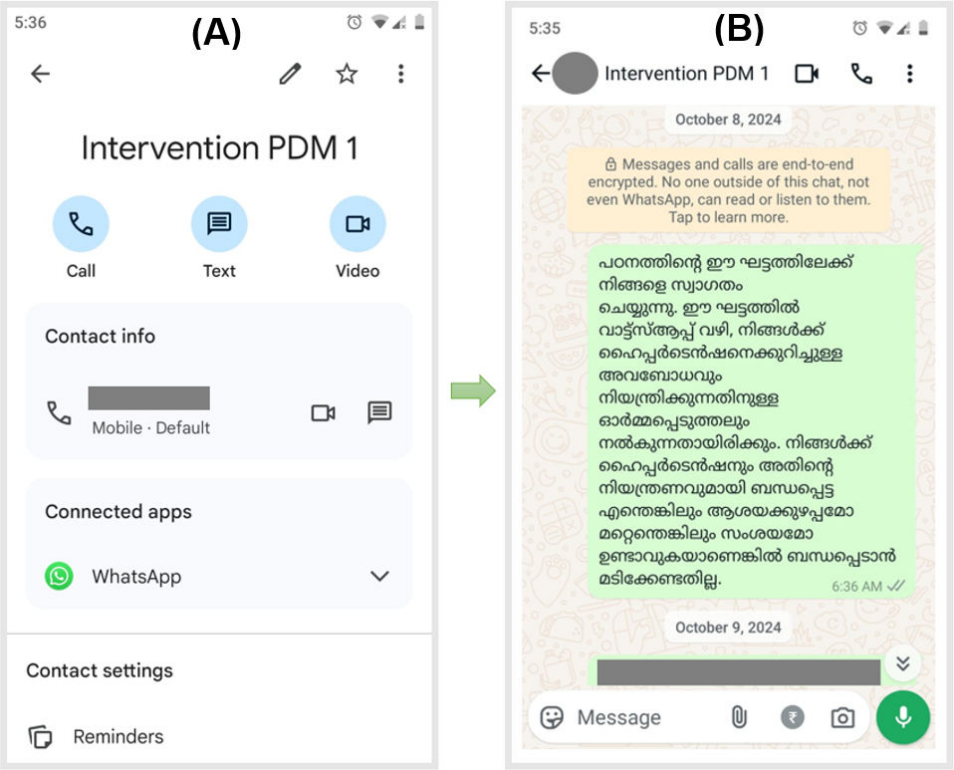
Saved contact and invitation to the participant for the intervention phase. (A) The contact is saved as “Intervention PDM 1” as this is the contact for participant 1 from the intervention group who is hypertensive (physician diagnosed and taking medication). (B) Chat page of participant 1. Translation of the message: “Welcome to this phase of the study. In this phase, you will be given awareness about hypertension and reminders to manage hypertension through WhatsApp. Contact us if you have any confusion or doubts related to hypertension and its management without hesitation.”

Participants in the intervention group will be advised to follow standard hypertension care. Additionally, participants will be invited to join the intervention through an invitation message and will be listed to receive personalized hypertension management messages via WhatsApp (Figures5  6). The health messages and posters will be shared in the morning at around 6 to 7 AM. Messaging will start within a month following the baseline survey. The intervention will continue from that day for the next 90 days. In the first month (first 30 days), messages will be shared daily; in the second month (middle 30 days), messages will be shared twice a week; and in the third month (last 30 days), messages will be shared once a week (7 days). There will not be a specific day of the week to send messages; days will be calculated from the first day that participants start receiving messages, not from the beginning of a month or a week. The messages will be shared in the participants’ preferred language (English or Malayalam), and their responses will be documented to check for participant engagement. The follow-up survey will be conducted after 3 months at the end of the intervention period. Participant engagement with the intervention will be actively monitored throughout the intervention period. A total of four steps will be followed as part of the audit trail to ensure that the messages are seen by the participants: (1) highlighted tick marks for each message shared with the participants, (2) message viewing status from the message information option on WhatsApp, (3) any response (in the form of a like or emoji) or comment from the participants after receiving the message or poster that was shared with them, and (4) monthly confirmation through phone call or home visit to ensure that the participants are receiving the messages. Any instances of disengagement from the participants will be noted and followed up on (through WhatsApp messaging, home visit, or phone call) by the principal investigator to reinforce the importance of being actively involved in the study and address any concerns.

**Figure 6.**
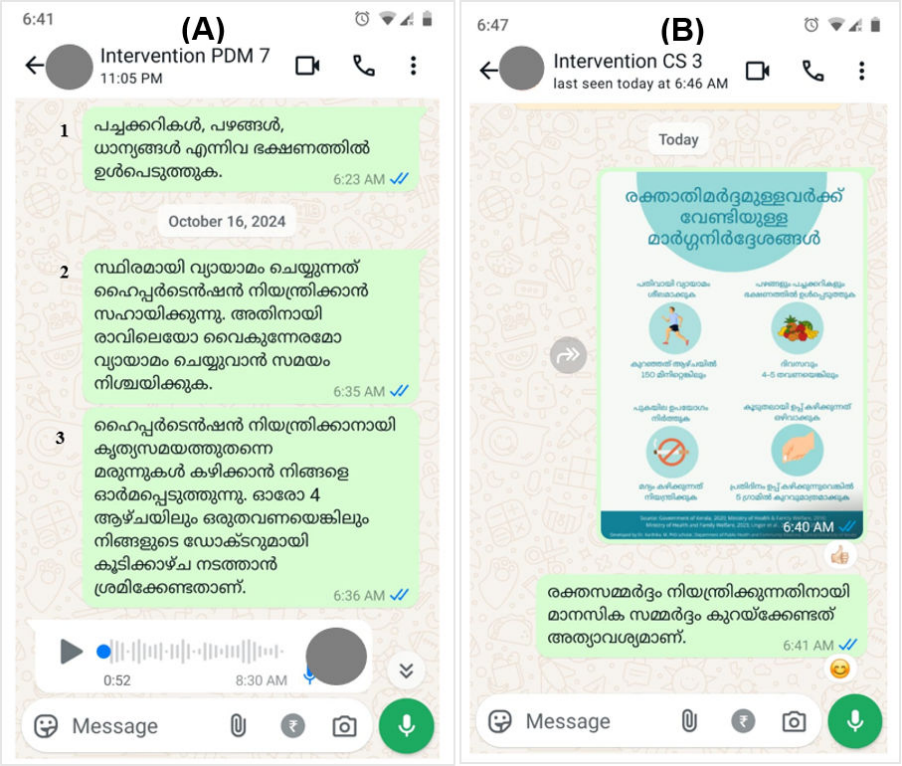
Participant-specific health messages related to healthy lifestyle and medication. (A) Translation of the messages from the chat page of Intervention PDM 7 (a physician-diagnosed participant with hypertension taking antihypertensive medication): “1) Include vegetables, fruits, and whole grains in the diet; 2) The practice of regular physical activities helps to control hypertension, so, fix a time for exercise in the morning or evening; 3) This is a reminder to take your anti-hypertensive medication on time. Try to have an appointment with your consulting doctor at least once every 4 weeks.” (B) Translation of the messages from the chat page of Intervention CS 3 (hypertension case found during screening): “Measures to control hypertension—practice exercise daily (at least 150 minutes a week), include fruits and vegetables in the diet (take at least 4-5 servings a day), restrict excess salt intake (consume only less than 5 gm), stop using tobacco, and control consuming alcohol” (from the poster) and “It is important to reduce mental stress to control hypertension” (from the message).

Participants in the control group will be advised to continue standard hypertension care and reminded of the follow-up survey 3 months after the baseline survey. To ensure that the participants in the control group also benefit from being part of the study, they will receive the messages and posters after the follow-up survey.

#### Data Collection Tools and Techniques

Prospective participants will be screened using a checklist. Blood pressure will be measured as per the guidelines of the World Health Organization and the American Heart Association [29,30] using an Omron HEM-7113 blood pressure monitor [31,32]. The responses to the questionnaire will be self-reported by the participants. Lifestyle factors (ie, diet, physical activity, and tobacco use) and medication adherence will be assessed before and after the intervention period. Lifestyle factors will be assessed through a series of questions on fruit and vegetable intake, salt consumption, processed food intake, physical activity, tobacco use, and alcohol consumption. Medication adherence will be assessed using the Hill-Bone Medication Adherence Scale and a self-developed questionnaire to assess the reasons for nonadherence to medication [33,34]. The *KoboToolbox* application will be used along with the pen-and-paper approach to collect survey data. Additional physical measurements, such as waist and hip circumference, will be taken using the Seca ergonomic retractable tape at baseline and follow-up [31,32,35]. Both the baseline and follow-up surveys will be conducted at the participants’ homes. Responses to WhatsApp messages will be recorded in a Microsoft Excel spreadsheet throughout the intervention period. All the tools (Table 1) are prepared in English, translated into Malayalam, and back translated into English. Loss to follow-up will be managed through revisits and prescheduled interviews based on the participants’ convenience except in cases of refusal, migration, or death.

**Table 1.** Data collection tools and techniques, along with the operationalization of variables.

Tool or technique and variable	Operationalization	References
Checklist for screening		
Eligibility factors	Age in years; self-reported pregnancy status, disability status, recent surgery, type 1 diabetes mellitus, and life-threatening diseases; access to a smartphone and WhatsApp via a “yes” or “no” question, and if yes, specify whose smartphone they have access to	[4,27,36]
Adapted questionnaires for the baseline survey		
Socioeconomic status	On the basis of the modified Kuppuswamy scale, a score of 26-29 indicates upper socioeconomic class, 16-25 indicates upper-middle socioeconomic class, 11-15 indicates lower-middle socioeconomic class, 5-10 indicates upper-lower socioeconomic class, and <5 indicates lower socioeconomic class	[37]
Sociodemographic characteristics	Self-reported sex, residence, marital status, educational level, occupation, living arrangements, monthly family income, number of children, and household members	[38]
Digital health literacy	Captured using a 6-item Likert-type questionnaire; based on the responses, a higher score denotes a higher digital health literacy	[39]
Intention to use the mHealth^a^ strategy	Captured using a 5-domain questionnaire with 20 items; based on the responses, a higher score denotes a higher intention to use the mHealth strategy for hypertension management	[30]
Physical measurements for screening and the follow-up survey		
Hypertension status	A physician-diagnosed case of hypertension and/or when blood pressure is measured and shows SBP^b^ ≥140 mm Hg and/or DBP^c^ ≥90 mm Hg	[40]
Hypertension emergency	When blood pressure is measured and shows SBP ≥180 mm Hg and/or DBP ≥110 mm Hg	[24]
Physical measurements for the baseline and follow-up surveys		
Waist circumference	Controlled waist circumference ≤90 cm for male individuals and ≤80 cm for female individuals	[35]
Waist-to-hip ratio	Controlled waist-to-hip ratio ≤0.90 for male individuals and ≤0.85 for female individuals	[35]
Adapted questionnaires for the baseline and follow-up surveys		
Awareness of hypertension	A total score will be calculated based on responses to 12 questions; the higher the score, the higher the awareness	[33]
Alcohol consumption and tobacco use	Responses of “yes” or “no”; if yes, then subsequent questions will be asked to assess frequency and pattern of alcohol consumption and the type of tobacco use	[40]
Processed food intake, fruit and vegetable intake, and salt intake	On the basis of the Nova classification system for processed food (based on level of processing) and adherence to the diet	[41,42]
Physical activity	Considered adequate when an individual has at least 150 min per wk of moderate activity or at least 75 min per wk of vigorous activity or an equivalent combination; the leisure-time physical activity will be considered adequate when the individual has at least 300 min per wk	[43]
Medication adherence status and reasons for nonadherence	On the basis of the Hill-Bone Medication Adherence Scale, the higher the total score, the poorer the adherence; in addition, the higher the score, the more reasons for nonadherence to medication	[33,34]
Treatment questionnaire for the baseline survey		
Status of health care service use for diagnosis, treatment, and others	Diagnosis of hypertension and comorbidities, duration of hypertension, frequency of blood pressure monitoring, type of health care facility for service, and status of telemedicine or mHealth service use	[38,40]
Treatment questionnaire for the baseline and follow-up surveys		
Details on antihypertension medication	Medication status, along with frequency and duration of medication; side effects of medication	[38,40]

a mHealth: mobile health.

b SBP: systolic blood pressure.

c DBP: diastolic blood pressure.

#### Validity and Reliability of the Tools and Measurements

The content and face validity of the tools were improved through literature and peer reviews. All the adapted questionnaires used for the study will be tested for internal consistency using Cronbach α values. Physical measurements and blood pressure measurements will be taken by the principal investigator of the study, who is a trained medical graduate with a Bachelor of Ayurvedic Medicine and Surgery and a Master of Public Health and currently pursuing a PhD in Public Health. Standard equipment and specific cutoffs will be used as per standard protocols for blood pressure [30], waist circumference, and waist-to-hip ratio measurement [35]. The electronic blood pressure monitor will be checked for accuracy using a calibrated sphygmomanometer from the health center.

#### Study Outcomes

The primary outcomes are the evaluation of the impact of the WhatsApp-led mHealth strategy on blood pressure measurements and adherence to medication and a healthy lifestyle in adults with hypertension aged between 40 and 59 years.

The secondary outcomes are the estimation of the prevalence and control of hypertension; percentage of participants adhering to antihypertensive medication; and prevalence of tobacco use, alcohol consumption, physical activity, diet patterns (such as frequency of fruit and vegetable consumption and processed food intake), and salt intake, as well as the estimation of digital literacy and the intention to use the mHealth strategy among adults with hypertension aged 40 to 59 years.

#### Statistical Analysis

The survey data collected will be entered into a Microsoft Excel spreadsheet and then exported to SPSS (version 26; IBM Corp) for coding and analysis. Codebooks and frequency tables will be generated to characterize the responses and identify any data entry errors. The missing data will be characterized as missing at random and missing not at random. The data missing at random will be addressed by creating a new variable or recoding an existing variable to account for the missing response. The data missing not at random will be addressed using the last observation carried forward approach (if the missing data were due to loss to follow-up) or regression imputation. Case deletion will be used if there are any missing data observed in multiple outcome variables. The analysis will be conducted adhering to the intention-to-treat approach. Study variables will be developed based on the operationalization plan shown in Table 1.

Descriptive analyses, including frequencies and percentages for categorical variables and means and SDs for continuous variables, will be conducted to understand the sample characteristics. The sociodemographic and health-related characteristics of the intervention and control groups will be compared at baseline. Furthermore, the outcome variables, including medication and lifestyle factors, will be compared between and within the intervention and control groups using baseline and follow-up data.

The comparison of categorical outcomes (medication and lifestyle factors) between and within the intervention and control groups will be conducted using the chi-square test and logistic regression. The regression analysis will be reported as unadjusted odds ratios with 95% CIs. The comparison of continuous outcomes (waist-to-hip ratio, awareness of hypertension, medication adherence score, and duration of physical activity) between the intervention and control groups will be conducted using a 2-tailed independent-sample *t* test (for normally distributed data) and the Mann-Whitney *U* test (for nonnormally distributed data). For comparisons of continuous outcomes within the intervention and control groups, a paired *t* test (for normally distributed data) and a Wilcoxon signed-rank test (for nonnormally distributed data) will be used.

The generalized estimating equations model will be used to assess the effectiveness of the mHealth intervention based on the longitudinal study data (data from the baseline and follow-up surveys). Variables such as time, intervention, time-intervention interaction, and other sociodemographic factors will be considered in the model. The analysis using the generalized estimating equations model will be presented as adjusted odds ratios with 95% CIs. The significance will be considered at a *P* value of less than .05.

### Qualitative Phase of the Study

#### Study Design

The qualitative phase will follow the phenomenological approach to deeply understand the lived experiences of participants during the period of the intervention.

#### Sampling

For the qualitative phase of the study, a quota sampling approach will be used among the participants in the intervention group. On the basis of adherence status (ie, high or low adherence after the intervention), participants with hypertension from the intervention group will be categorized into subgroups using a checklist. Medication adherence, as well as adherence to lifestyle modifications (restriction in salt, control of processed food intake, adequate fruit and vegetable intake, and adequate physical activity), will be considered for categorizing participants as having high or low adherence. Sample selection will continue until data saturation is reached.

#### Data Collection

Face-to-face in-depth interviews will be conducted using an in-depth interview guide developed based on existing models for health care use, medication adherence, behavior adaptation, technology acceptance, and use of mHealth for adherence. The qualitative data will be captured as field notes and voice recordings. The in-depth interviews will be conducted at a location that is convenient for the participants and without the presence of any nonparticipants. The in-depth interview guide will be prepared in English, then translated into Malayalam, and then back-translated into English. All the interviews will be conducted in Malayalam (the vernacular language in the region).

#### Study Outcomes

The primary outcome is the in-depth understanding of the participants’ views on feasibility, facilitators, and challenges of the adoption of the mHealth strategy for achieving adherence.

The secondary outcomes are insights into the facilitators and challenges of adherence to medication and a healthy lifestyle.

#### Data Analysis

The qualitative data from the in-depth interviews will be analyzed using a thematic approach. The in-depth interview data will be translated and transcribed, and then the transcripts will be read and reread for familiarization. Deductive coding will be conducted based on the aforementioned existing models, and an inductive coding strategy will be adopted when new factors emerge from the in-depth interview transcripts. At least 2 researchers will code and generate themes to explain the phenomena and facts. Member checking with the interview participants and peer debriefing will be conducted to improve the trustworthiness of the qualitative findings. The qualitative analysis software NVivo (version 12; Lumivero) will be used to facilitate coding and theme development.

### Ethical Considerations

The study received ethics approval (IHEC/CUK/2024/04) from the Institutional Human Ethics Committee of the Central University of Kerala on August 1, 2024. Informed consent from participants will be obtained upon their enrollment in the study. A personal identification number will be used to ensure the privacy of the participants. The recruited participants will be allowed to withdraw from the study at their request irrespective of the stage at which they are in the study. In case of any unforeseen medical emergencies, the participants will be guided to receive essential treatment from the nearest public health care facility. All the participants from the intervention and control groups will benefit from the intervention at the end of the study. The participants will not receive any financial or material compensation for taking part in the study.

### Clinical Trial Registration

The study was registered as a nonrandomized active-controlled trial (phase 1 and phase 2 combined) in the Clinical Trials Registry – India. This study protocol version was approved and received the registration number CTRI/2024/09/073166 on September 2, 2024.

## Results

All 240 participants were recruited by July 2025. The study completed data collection in August 2025 and is expected to finish data analysis by April 2026. The study results are expected to be submitted for publication in July 2026. The baseline survey will help understand the participant characteristics, medication status, lifestyle factors, and blood pressure measurements before the intervention. The intention to use the mHealth strategy for hypertension management is also measured at this stage. The follow-up survey data will provide the participant characteristics after the intervention phase. The baseline and follow-up survey data will allow us to study the effectiveness of the intervention on the medication and lifestyle variables. Furthermore, the in-depth interviews will help provide insights into the lived experiences of participants during the period of the intervention, focusing on the adaptation of the mHealth intervention for adherence to medication and a healthy lifestyle. The interviews will also enable the understanding of the perceived effectiveness of the intervention. The study has not received any funding.

## Discussion

### Expected Findings

The study will develop an mHealth strategy to improve medication and lifestyle adherence for hypertension management. WhatsApp was considered a suitable platform as it is cost-effective and user-friendly. Moreover, it does not need to be separately downloaded and subscribed to like other apps as most people already use it for regular messaging [10,11].

The study seeks to establish a proof of concept for a WhatsApp-led mHealth strategy that can be integrated into hypertension management at the community level. This strategy could function as a means for timely reminders and encourage individuals to change their behavior, improving medication adherence and lifestyle modifications [40]. Similar approaches could be adopted for individuals with other chronic noncommunicable diseases. Moreover, health care professionals, especially frontline health workers, can contact, provide information to, and receive updates from patients through WhatsApp as instant messaging platforms such as this one can facilitate mHealth services by acting as a medium for communicating health advice and hypertension management guidelines [5,6,9,12,13].

Selecting only 1 taluk from the Kannur district is a major limitation. However, considering that there are similarities in the sociodemographic and economic conditions, linguistic homogeneity (the predominant language is Malayalam), and commonalities in the political and administrative factors, the findings could be generalized to the context of Kerala as a whole. The study adopted a quasi-experimental study design with an intervention group and a control group because the main aspects of a randomized controlled trial, such as randomization, matching, and blinding, cannot be applied in this context. Unlike other trials, this study uses an mHealth intervention, which acts as a platform for sending hypertension awareness messages and reminders for a healthy lifestyle and medication adherence in addition to the routine hypertension care available in health centers. There could be limitations in establishing causal conclusions about the observed effects. However, the qualitative interviews with the participants who received the intervention could help obtain a deeper understanding of their experiences with and adoption of the intervention to adhere to a healthy lifestyle and medication.

The follow-up survey time point of 3 months after the intervention was determined based on the review period (ie, 3 months) for lifestyle and blood pressure measurement changes as per the hypertension management protocol of the Department of Health Services, Government of Kerala [24]. This duration of 3 months is also based on the minimum period required for behavior changes or to develop new habits, including physical activity and dietary changes [12,44,45]. The long-term sustainability of behavior change and the durability of the mHealth intervention will be explored through the qualitative interviews. Even though the intervention ends after 3 months, the ability to keep the messages for an indefinite time, as well as the option to create a backup in WhatsApp, allows the participants to go through the messages again if needed after the intervention period.

WhatsApp is a ubiquitous platform that is not primarily meant for medical communication. However, given the real-time nature of WhatsApp-based intervention and health messaging specific to the individual participant, this study is expected to be effective in improving adherence to medication and healthy lifestyle and have better participant engagement with the mHealth intervention.

The individualized delivery method, with a person-to-person (researcher-to-participant) messaging system rather than a WhatsApp group or community, will help minimize contamination between the intervention and control groups. Additionally, all participants will be recruited from their homes and monitored regularly during the follow-up period until the end-line survey. In addition, during sample size estimation, the researcher accounted for the cluster effect to obtain an adequate sample size to prevent the reduction in the samples’ power due to contamination.

The findings from the study will contribute to improving the planning and implementation of mHealth services for managing chronic diseases such as hypertension in India and other similar settings. Tailoring health messages specifically to the individuals could be challenging. However, prestructuring the messages will be helpful for easy dissemination. The researcher will recruit 4 batches of approximately 30 participants each into the control and intervention groups and then start sharing the messages individually with the participants in the intervention group. The entire intervention will be completed in these 4 batches. This is only for the convenience of the participants and the researcher to reduce the gap between the baseline survey and the first day of the intervention. The end-to-end encryption option of WhatsApp helps protect each individual’s chat data, and saving the participants’ contact information using code names will help protect their privacy.

### Dissemination Plan

The findings from the study will be presented at national and international conferences and published in peer-reviewed journals. A study report will be submitted to the Central University of Kerala library, the Institutional Human Ethics Committee of the Central University of Kerala, and the Clinical Trials Registry – India. In addition, study summaries will be provided to the officials of the administrative units selected for the study.

### Conclusions

The core focus of our study is to analyze both quantitatively and qualitatively the impact of a WhatsApp-led mHealth strategy on blood pressure measurements and adherence to medication and a healthy lifestyle in adults with hypertension aged between 40 and 59 years. The intervention is expected to improve hypertension control among study participants. Additionally, the participants are expected to have increased awareness, adherence to medication, healthy behaviors, and regular follow-ups or monitoring of blood pressure. The study will also help understand the participants’ acceptance of the WhatsApp-led mHealth strategy for the self-management of hypertension.

## Abbreviations

mHealth: mobile health
